# Integrating social identity theory and family systems theory to explain how sociocultural norms become family life

**DOI:** 10.3389/fpsyg.2026.1902682

**Published:** 2026-08-24

**Authors:** Durvashi Mathoora, Kwan Foong Chee, Serena Leow, Chin Ong Woon, Long Chiau Ming

**Affiliations:** 1 Faculty of Medical and Life Sciences, School of Psychology, Sunway University, Sunway City, Malaysia; 2 Faculty of Arts and Social Sciences, Sunway University, Sunway City, Malaysia; 3 Sunway Business School, Sunway University, Sunway City, Malaysia; 4 School of Pharmacy, Sunway University, Sunway City, Malaysia; 5 Department of Pharmacy Practice, Faculty of Pharmacy, Universitas Airlangga, Surabaya, Indonesia

**Keywords:** family rules, family systems theory, IPA, Mauritius, qualitative methodology, reputational networks, social identity theory, sociocultural norms

## Abstract

A persistent challenge in cultural family psychology is explaining how publicly circulating expectations about respectability, marriage, achievement, and filial duty become rules, resource conditions, and emotional climates within particular households. This theoretical and methodological article develops a bounded integration of Social Identity Theory (SIT) and Bowen’s Family Systems Theory (FST). The model specifies four linked propositions: sociocultural norms acquire identity and reputational significance; family members with unequal authority and resources translate those stakes into domestic rules and responses; the relational effects of those responses depend on negotiability, material dependence, and structural position; and family experiences can reproduce, soften, or contest the original norm. Three extended illustrations from an Interpretative Phenomenological Analysis (IPA) study of five parents and five adult children in Mauritius examine conditional support, an age-linked adulthood checklist, and marriage endogamy. Each illustration combines convergent material with counter-examples or qualifying cases and integrates gendered authority, classed access to resources, ethnoreligious boundary-making, and heteronormativity into the analysis itself. The article also documents participant characteristics, recruitment, interview procedures, idiographic analysis, negative-case decisions, reflexive safeguards, and an extract-to-theme audit trail. The excerpts establish the analytic plausibility and limits of the framework; they do not demonstrate prevalence, causality, or validation of a universal model. The proposed integration is therefore offered as a context-sensitive analytic strategy requiring fuller empirical and comparative testing.

## Introduction

Cultural family psychology faces a recurring theoretical problem. Social-psychological approaches explain how shared expectations are internalised, why they acquire motivational force, and why deviation may trigger stigma or sanction (Bicchieri and Mercier, 2014). Family-psychological approaches explain how rules, roles, and relational patterns organise daily domestic life. These bodies of work are often discussed separately. Research on norms, including Social Identity Theory (Tajfel and Turner, 1979), does not always specify what happens when public expectations enter the family home. Conversely, Family Systems Theory (Bowen, 1978) describes interactional processes but does not by itself explain why particular household rules carry strong cultural and reputational stakes.

The analytic problem lies in the translation between these levels: how a community expectation becomes a household rule, how anticipated public judgement becomes a condition attached to support, and how a prototype of the “good family” becomes a private standard for evaluating relationships. This article treats SIT and FST as potentially complementary rather than inherently or uniquely so. Their value must be shown by specifying the mechanism that connects the levels, identifying what each theory adds, testing interpretations against counter-cases, and locating unequal authority and material resources within the mechanism rather than outside it.

This is a theoretical and methodological article, not a primary empirical report. The empirical material is drawn from a completed IPA study of parent-adult child relationships in Mauritius and is used to examine whether the combined lens can generate interpretations that remain accountable to participants’ language, local narrative context, alternative readings, and disconfirming material. The evidentiary claim is deliberately limited: the excerpts illustrate analytic operations and boundary conditions; they do not establish the prevalence, causal direction, or general validity of the proposed pathway.

Mauritius is an analytically useful setting because it is a small, plural society shaped by multiple ethnic, religious, linguistic, and migration histories. Kinship, neighbourhood, and religious or community networks can make private choices socially visible, while family expectations are negotiated amid changing educational, occupational, and economic conditions (Eisenlohr, 2006; Eriksen, 1998; Sambajee, 2016). This does not make Mauritian families uniform or imply that reputational dynamics are uniquely intense. It makes the public-private interface visible while also requiring attention to within-context variation by class, gender, sexuality, ethnicity, religion, migration history, living arrangement, and access to resources.

The paper has four objectives: (1) to specify a recursive and power-sensitive pathway through which public normative expectations acquire identity and reputational stakes and are translated into household interaction; (2) to show, through extended empirical illustrations, what the dual lens adds beyond either theory alone and where the data do not support a theoretical inference; (3) to make the IPA procedure sufficiently transparent for readers to assess how extracts became interpretative claims, including the handling of negative cases, and (4) to state the present constraints on the validity and scope of the argument rather than treating limitations only as future research opportunities.

The paper first presents each theory and its limits, then specifies the integration, its propositions, and its safeguards. Three illustrations address conditional support, the adulthood checklist, and marriage endogamy. The final sections document the primary study and analytic audit trail, explain the role of theory within IPA, and delimit what the current evidence can and cannot sustain.

## Part I: the theories separately

### Social identity theory: the reputational economy

Social Identity Theory proposes that part of self-definition derives from group memberships. Group prototypes—shared understandings of how a group member should think, feel, or act—become psychologically relevant through categorization and identification, while social comparison shapes the pursuit of favourable group standing (Hogg, 2006; Tajfel and Turner, 1979; Turner et al., 1987). Although developed principally to explain intergroup processes, the social identity approach has also been applied to organisational, health, and cultural contexts (Haslam et al., 2018; Reicher et al., 2010).

Applied to family and cultural norms, SIT offers three contributions. First, it helps explain why community expectations may feel personally compelling rather than merely external: group prototypes can become part of how people understand themselves and their obligations (Ellemers et al., 2002). For a parent who understands endogamous marriage as part of group continuity, encouraging an in-group match may therefore be experienced as identity-consistent care rather than simple compliance with outside pressure.

Second, SIT identifies the comparative dimension of normative pressure. Expectations are interpreted against reference groups whose behaviour and perceived success provide standards for evaluating one’s own family. The familiar concern with “what people will say” can therefore be analysed as anticipated audience judgement and monitoring of relative standing within a reputational field (Festinger, 1954; Tajfel and Turner, 1979). In community-embedded settings, the proximity of these audiences may make comparison especially difficult to ignore.

Third, SIT clarifies why non-conformity can be experienced as more than personal disappointment. Deviation from a valued prototype may threaten positive distinctiveness—the sense that one’s group compares favourably with relevant others—and may therefore be interpreted as reputational damage to the family or community (Tajfel and Turner, 1979). The strength of this interpretation, however, remains an empirical question rather than a universal feature of family life.

SIT is less specific about domestic enactment. Knowing that a marriage or achievement norm carries reputational weight does not explain who communicates it, how rules are negotiated, how support is allocated, what happens when expectations are contested, or why families exposed to similar norms respond differently. These are interactional and systemic questions.

### Family systems theory: the domestic mechanism

Bowen’s (1978) Family Systems Theory conceptualises the family as an emotional unit with recurrent patterns of interaction and mechanisms for managing stability and change. Relevant concepts include differentiation of self, triangulation, emotional cutoff, and multigenerational transmission (Bowen, 1978; Kerr and Bowen, 1988). Structural family concepts such as roles, boundaries, and coalitions are also useful for describing how expectations are organised in everyday family life (Minuchin, 1974).

First, FST provides a vocabulary for specifying how norms become household practices: through explicit and implicit rules, the assignment of roles, the distribution of decision-making authority, and the management of boundaries. It therefore shifts analysis from the existence of a norm to the interactional processes through which it is maintained or revised.

Second, FST treats pressure as systemic rather than individual. When one family member seeks greater autonomy, other members may alter monitoring, advice, alliances, or emotional distance. The concept of homeostasis captures the tendency of a family system to restore familiar patterns when change is experienced as threatening, although stability should not be assumed to be equitable or beneficial for every member.

Third, FST helps explain variation across families living within the same normative field. Families differ in their tolerance of disagreement, flexibility of boundaries, and capacity to maintain connection while permitting individual difference. Such variation is central to understanding why similar cultural expectations may be experienced as guidance in one family and as constraint in another.

FST is less equipped to explain why particular domains acquire their cultural intensity. It can describe boundary tightening or coalition-building around an adult child’s partner choice, but it does not fully explain why marriage may be interpreted as a public marker of group continuity and family standing. SIT supplies concepts for analysing those identity and reputational stakes.

## Part II: the integration—from public reputation to private interaction

### The complementarity argument

The case for integration rests on adjacent but non-equivalent levels of analysis. SIT identifies the public and communal processes through which some expectations become identity-relevant, comparatively monitored, and reputationally consequential. FST identifies the household processes through which family members respond to those perceived stakes by arranging rules, decision-rights, alliances, boundaries, emotional distance, and access to resources. The integration is useful only when it traces movement between these levels without treating either public identity or family stability as a sufficient explanation.

The proposed translation mechanism is recursive. Publicly circulating expectations provide prototypes of the “good family”, “respectable parent”, or “successful adult”. Identification and social comparison may invest those prototypes with reputational stakes. Those stakes do not enter a neutral household: family members differ in authority, control of housing or money, social legitimacy, and exposure to sanction. Domestic calibration therefore occurs through unequal capacities to define acceptable action, request explanations, attach conditions to support, mobilise relatives, or withhold disclosure. The resulting experiences of care, recognition, vigilance, conflict, or negotiated autonomy can reinforce the norm, loosen its domestic enforcement, or make the norm itself an object of critique.

Table 1 maps the pathway. The stages are analytic rather than strictly sequential, and a missing link is theoretically meaningful. A public norm may be visible without becoming identity-salient; reputational concern may exist without household enforcement; a rule may be present without conditional belonging, and disagreement may lead to accommodation rather than homeostatic tightening.

**Table 1:** Power-sensitive conceptual pathway of SIT-FST integration.

Analytic stage	SIT contribution	FST contribution	Integrated and power-sensitive implication
Public normative field	Group categorization, prototypes, social comparison, and anticipated audiences.	External expectations enter the family emotional field but do not determine a single response.	A norm becomes analytically relevant only when participants orient to it as identity- or reputation-bearing; absence, rejection, or indifference is disconfirming evidence.
Differential stakes and authority	Positive distinctiveness and belonging may be unevenly attached to gender, sexuality, ethnicity, religion, and class position.	Family members occupy unequal roles and control different resources, alliances, and decision-rights.	Ask who defines respectability, whose deviation is most visible, who can enforce a rule, and who bears the material and relational costs.
Domestic calibration	SIT identifies what may be symbolically at stake but does not specify enactment.	Advice, monitoring, rules, triangulation, emotional tone, boundaries, and resource allocation organise the response.	Reputational stakes become interaction only through observable or narrated practices. A theoretical label is insufficient without a verbatim and contextual anchor.
Relational outcomes	Conformity or deviation can affect identity affirmation and perceived standing.	Patterns may tighten, relax, fragment, or be renegotiated; connection and autonomy can coexist.	The same practice can be care, leverage, or ambivalent involvement depending on contingency, negotiability, dependence, and participants’ meanings.
Feedback and revision	Successful deviation or changing reference groups may alter prototypes and comparison standards.	Families may revise rules after conflict, demonstrated responsibility, or changing dependencies.	Feedback can reproduce, soften, or contest a norm. Cross-sectional accounts can suggest this process but cannot directly establish temporal causality.

Structural relations are not an external “context” added after analysis. They shape the visibility of norms, the distribution of authority and resources, the available forms of resistance, and the consequences of disagreement at every stage.

### Analytic propositions and boundary conditions

*Proposition 1—Norm salience is contingent*. A culturally available expectation becomes explanatory only when the participant or family treats it as relevant to identity, standing, belonging, or obligation. Accounts of indifference, explicit rejection, or low pressure limit the proposition rather than being treated as noise.

*Proposition 2—Translation requires a domestic mechanism*. Reputational concern cannot be inferred to shape family life unless it is connected to narrated practices such as repeated questioning, comparison, rule-setting, shifts in emotional tone, alliance-building, or conditions attached to resources.

*Proposition 3—Power conditions the mechanism*. The ability to convert a preference into a rule depends on authority, material dependence, social legitimacy, and access to supportive allies. The model therefore distinguishes mutual interdependence from leverage and family stability from equitable family functioning.

*Proposition 4—Feedback is not synonymous with restoration*. Family responses may tighten an inherited pattern, but they may also broaden a timeline, accept an exception, redefine good parenting as guidance rather than command, or shift disclosure and distance. These alternatives are part of the model, not deviations from it.

### Critical safeguards

Three safeguards govern the analysis. First, interpretation must be power-critical. Describing identity protection or systemic stability without asking whose interests are served can naturalise father-centred authority, heteronormative partnership scripts, ethnoreligious boundary policing, and unequal control of housing, money, or mobility (Connell, 2005; Hare-Mustin, 1987; Ridgeway, 2011). Every illustration therefore identifies the resource or authority relation through which a norm can be enforced and states when the available material cannot support a subgroup comparison.

Second, the integration must remain empirically defeasible. SIT and FST are used to formulate questions, not to supply answers in advance. A proposed interpretation is retained only when it is anchored in the participant’s wording, the local narrative context, and a documented relational process; it is revised or set aside when a counter-case makes a narrower explanation more credible.

Third, the integration must remain context-sensitive without essentialising context. Mauritius is not treated as a homogeneous “collectivist” case. The analysis attends to plural ethnoreligious histories, small-scale social visibility, intergenerational involvement, migration, and economic change while preserving differences among families and limiting transferability to analytically comparable conditions (Sam and Berry, 2016).

## Part III: extended illustrations from the empirical record

The following illustrations were selected from completed idiographic and cross-case theme tables because they make the proposed translation process visible and because each domain contains material that complicates a one-directional account. The quotations are therefore presented in short analytic sequences rather than as isolated proof-texts. Each illustration includes: a focal process, a counter-example or boundary condition, an integrated reading, a power and structural analysis, and a statement of what the material cannot establish. This structure increases empirical accountability but does not turn the article into a full findings report.

### Illustration 1: conditional support as recognition, resource allocation, and negotiated dependence

In the primary study, “Conditional vs. Open support” emerged as a sub-theme. Conditional support refers here to practical, financial, or emotional help that is experienced as more secure, affirming, or readily available when an adult child remains within an accepted range of choices. The definition requires a narrated contingency or patterned shift; support is not coded as conditional merely because a parent sets a rule or expresses concern.

*“Parents who are supportive financially… but you kinda are… wired… to seek their approval.” (AC1)*

AC1’s account holds care and regulation together. The descriptive content is reliable financial support; the linguistic feature is “wired”, a metaphor that presents approval-seeking as internalised and partly automatic; the experiential consequence is self-monitoring. SIT draws attention to approval as identity-relevant recognition: maintaining the position of a responsible or respectful family member may matter beyond the practical value of the support. FST draws attention to how approval becomes organised through recurring interactions—anticipated reactions, careful disclosure, comparison, and the emotional tone surrounding assistance. The dual reading therefore links a reputationally meaningful identity position to a domestic pattern without claiming that financial support was explicitly threatened.

*“We’ve been a household where… we are very open.” (P1)*

P1 provides a counter-case within the same domain. Support is described as a standing resource rather than a reward for strict conformity. The case prevents interdependence from being equated with conditionality: close family involvement can include explanation, provisional choices, and continued belonging. Analytically, the contrast changes the question from whether parents help to how the meaning, tone, and negotiability of help are experienced. It also limits a homeostasis-only reading, because family continuity can be maintained through openness rather than tightening.

*“As long as they stay in my house, they will not be able to… do as they wish.” (P5)*

P5’s statement makes the resource relation explicit: authority is anchored to housing. Yet the quotation is not, by itself, evidence of conditional belonging or withdrawal of support. It becomes relevant to conditionality only when the rule is connected to consequences for security, recognition, or access to resources. This distinction is a critical safeguard against treating every boundary as coercion. At the same time, the statement shows why power cannot be separated from systemic analysis. A co-resident adult child may have less practical capacity to refuse a rule than a financially independent adult, and the same “family preference” therefore carries different force depending on housing alternatives, income, and the availability of supportive allies.

The integrated interpretation is bounded. These excerpts demonstrate that approval, help, and household authority can become linked, and that open support provides a disconfirming alternative. They do not establish how frequently support was withdrawn, whether parents intended leverage, or whether conditionality differs systematically by sex, class, ethnicity, or religion. The defensible claim is narrower: the framework helps distinguish supportive interdependence, implicit approval-contingency, and materially backed authority when the participant’s account supplies the relevant evidence.

### Illustration 2: the adulthood checklist as public standard, household clock, and classed demand

The adulthood checklist refers here to a culturally shared, often age-linked set of milestones—completing education, obtaining stable work, acquiring property or mobility, and marrying—through which adult competence and readiness are publicly interpreted. The term describes a normative sequence; it does not imply that every family endorses the same milestones or enforces the same timetable.

*“…we would like to see our children completing their studies, and of course applying for a job… at 25, we already expect…” (P3)*

P3’s “of course” and “at 25” make the normative clock visible. SIT highlights the taken-for-granted comparative standard through which adulthood becomes legible to relatives and community audiences. FST highlights how the public timetable can organise domestic interaction: the timing and repetition of questions, the pace of advice, and decisions about continuing financial support. The relevant mechanism is not age alone but the conversion of an age-indexed prototype into recurring household expectations (Table 2).

**Table 2:** Empirical accountability across the three illustrations.

Illustration	Convergent material	Counter-case or boundary condition	Bounded inference
Conditional support	AC1 links reliable financial help with internalised approval-seeking; P5 locates authority in housing.	P1 describes open support; a house rule alone is not coded as conditional support.	The framework distinguishes care, implicit contingency, and materially backed authority; it does not establish intentional withdrawal or prevalence.
Adulthood checklist	P3 and AC5 describe age-indexed milestones; P1 identifies an inherited status model.	AC3 reports little pressure; families may broaden timelines and routes.	Public milestones can become household clocks, but translation is contingent and structurally constrained.
Marriage endogamy	AC2, AC4, and AC3 describe ethnoreligious, arranged-marriage, and heteronormative pressure.	Negotiated exceptions, deferral, and uneven authority within households complicate uniform enforcement.	Marriage may concentrate identity stakes and family mobilisation; no universal gender or community pattern is claimed.

*“I think all parents want their kids to succeed… we are still mostly stuck with the old model… having people who have good jobs… secure, well-paid, and with some kind of social status recognition.” (P1)*

P1 names both the attraction and rigidity of the model. Security and status can be understood as care for the adult child and as evidence of family competence, while “stuck” signals recognition that inherited standards may not fit current routes through education and work. This account supports the recursive element of the model: family members can identify a prototype, orient to it, and simultaneously question its adequacy.

*“I’m 26 years old now. I’m expected obviously to have a driving licence, to have a house… To be married… to have my life together.” (AC5)*

AC5 compresses material, relational, and moral achievements into one measure of readiness. “Obviously” suggests a socially recognisable standard, while “have my life together” shows how missing milestones can become an evaluation of the person rather than a description of circumstances. The power-sensitive reading is classed and structural. Housing prices, access to credit, employment insecurity, tuition costs, transport needs, and migration opportunities determine whether the list is achievable. If these conditions are omitted, SIT-FST risks translating a constrained labour or housing market into individual inadequacy or family dysfunction.

*“I don’t feel very pressured about anything.” (AC3)*

AC3 is a disconfirming case against the assumption that socially available milestones necessarily produce intense household pressure. It indicates that clear decision-rights, flexible timelines, or family acceptance can interrupt the proposed translation pathway. The model therefore predicts variation: a public checklist may remain culturally intelligible without becoming a tightly enforced household clock.

The illustration supports a bounded claim: age-linked prototypes can organise family conversation and recognition, but their domestic force depends on family flexibility and material conditions. The current excerpts cannot establish whether a particular class group experiences more pressure, whether delayed milestones cause distress, or whether the checklist operates uniformly across Mauritian communities. Those questions require comparative and longitudinal evidence.

### Illustration 3: marriage endogamy as ethnoreligious boundary-making, heteronormativity, and family mobilisation

Marriage endogamy is defined here as an explicit or implicit expectation that an adult child choose a partner from the same ethnic or religious community. In the primary study, this expectation sometimes intersected with arranged-marriage pathways and heteronormative assumptions about partnership and children. The analysis does not treat endogamy as a uniform Mauritian norm; it examines how some participants narrated its symbolic and interactional force.

*“…pressure about marrying someone… of the right ethnic community…” (AC2)*

The adjective “right” marks partner choice as a boundary of legitimate belonging rather than a purely private preference. SIT clarifies how marriage may be read as a visible statement about group continuity, ritual participation, and family standing. The account also shows that identity categories are not neutral: defining one community as the correct marital boundary gives some relationships legitimacy and positions others as reputational risks.

*“They really started pestering me… for an arranged marriage.” (AC4)*

AC4 identifies the interactional form of enforcement. “Pestering” conveys repeated, low-intensity insistence rather than a single prohibition. FST directs attention to the cumulative pattern: recurring reminders, the possible recruitment of relatives, selective disclosure, delay, and the search for a more flexible family ally. The dual lens explains why the interaction persists—the domain carries symbolic stakes—and how those stakes become a family campaign rather than remaining an abstract community value.

*“Be straight, have kids, be happy… the expectation is so high…” (AC3)*

AC3 exposes the heteronormative structure of the marriage script. Sexuality, fertility, and happiness are compressed into a single socially approved life course. A power-critical analysis therefore asks not only how a family maintains an in-group boundary but whose relationships are rendered unintelligible or costly by that boundary. The symbolic protection of continuity can translate into unequal recognition, pressure to conceal, or reduced room to negotiate for adults whose identities or partnerships do not fit the script.

*“My mom… trusts me a lot… but my dad?… he had some expectations on me…” (AC5)*

Although this quotation is not specific to marriage, it demonstrates that authority can be unevenly distributed within one household. Maternal trust and paternal expectation create different routes for disclosure and negotiation. This supports a family-systems analysis of alliances and differential decision-rights while also locating gendered authority within the household. It does not, however, justify a general claim that daughters face more severe marriage enforcement than sons; the small purposive sample was not designed for that comparison.

Complicating material in the primary analysis included negotiated exceptions, temporal deferral, and families that accepted a partner across boundaries when character, stability, or mutual respect were treated as more important. These cases show that ethnoreligious boundaries can be reinterpreted rather than simply obeyed or ruptured. The defensible inference is that marriage can carry concentrated identity stakes and mobilise repeated family responses in some cases. The material cannot establish prevalence, a single gender pattern, or the causal effect of endogamy pressure on wellbeing.

## Part IV: methodological transparency and implications for IPA-based research

The proposed SIT-FST integration was developed in relation to a primary study analysed using Interpretative Phenomenological Analysis (IPA; Smith et al., 2009). Because the empirical illustrations are central to evaluating the framework’s analytic plausibility, the study design and interpretative procedure are reported here in sufficient detail for readers to assess where the claims came from and where theoretical imposition was constrained (Table 3).

**Table 3:** Participant characteristics and interview details.

Pseudonym	Age/sex	Ethnicity	Religion/background	Interview
Adult children				
AC1	31/Male	Indo-Mauritian	Muslim	47 min 09 s
AC2	31/Female	Indo-Mauritian (Bihari, Tamil, Gujarati)	Muslim/agnostic (non-practising)	47 min 29 s
AC3	28/Female	Mixed	Christian/apostate	27 min 20 s
AC4	32/Female	Indo-Mauritian	Hindu	36 min 47 s
AC5	26/Male	Creole	Agnostic/Catholic	47 min 53 s
Parents				
P1	49/Female	Mixed (Chinese father; Mauritian mother from Muslim family)	Atheist (baptised Catholic)	55 min 34 s
P2	65/Female	Indo-Mauritian	Hindu	43 min 09 s
P3	55/Male	Indo-Mauritian	Sunni Muslim	23 min 59 s
P4	72/Male	Indo-Mauritian	Hindu	28 min 24 s
P5	55/Female	Indo-Mauritian	Hindu	52 min 17 s

### Study design, recruitment, and participants

The primary study used a qualitative phenomenological design. Participants were recruited in Mauritius through purposive, criterion-based sampling with maximum variation on gender and ethnoreligious background. An online screening form was distributed through Instagram, Facebook, and WhatsApp. Adult-child participants were aged 18 or older and had lived in Mauritius during approximately ages 1–17; parent participants had an adult child who had lived in Mauritius during that developmental period. Sufficient English proficiency, informed consent, and willingness to be recorded were required. Recruitment concluded at 10 interviews—five adult children and five parents—because the focused sample and in-depth accounts provided sufficient information power for idiographic analysis, while the final interviews continued to add nuance rather than substantively new codes (Malterud et al., 2016).

Each participant completed one in-depth, one-to-one semi-structured interview via Microsoft Teams. Interviews lasted approximately 24–55 min and were conducted in English. The guide asked broad questions about common Mauritian expectations, their influence on parent-adult child interactions, relational pressures, mental wellbeing, coping, and desired social change. Follow-up prompts requested concrete episodes and clarified whether expectations were communicated directly or indirectly. SIT and FST informed sensitising areas of attention, but participants were not presented with theoretical terminology or asked to confirm theoretical propositions. Ethical approval was granted by the Sunway University Research Ethics Committee (2024/REC0130).

### Idiographic analysis and the extract-to-theme audit trail

Analysis followed an idiographic IPA sequence. Each transcript was read several times, with selected audio segments revisited for tone and affect. Descriptive notes recorded what was said; linguistic notes attended to metaphors, pauses, hesitations, and emphasis; conceptual notes developed tentative interpretative possibilities. Emergent themes were retained only when they remained accountable to a verbatim anchor, the local narrative context, and a short analytic gloss explaining what the theme was understood to do in that case. Each case was completed and summarised before the next was opened. Cross-case matrices were constructed only after all 10 idiographic analyses, and candidate shared themes were formed when a similar relational process appeared across cases without erasing meaningful divergence.

AC1 provides a concrete audit trail. For “Parents who are supportive financially… but you kinda are… wired… to seek their approval,” the descriptive note recorded reliable care alongside approval-seeking; the linguistic note identified “wired” as a metaphor of internalised expectation and the pauses as ambivalence; and the conceptual note interpreted approval-contingent support as increasing self-monitoring. The provisional case-level phrase “support tied to approval” was then considered alongside other AC1 extracts about careful speech and fear of disappointing parents. Only after the complete within-case analysis did this contribute to the cross-case sub-theme “Conditional vs. Open Support”. The idiographic meaning—internalised vigilance within reliable care—was retained rather than replaced by a generic theory code.

Microsoft Word supported close annotation, Excel retained extracts, transcript locations, analytic glosses, within-case clusters, and cross-case matrices, and NVivo was used supplementarily for retrieval and keyword coverage checks. Software did not generate themes or substitute for contextual re-reading.

### Negative cases and disconfirming evidence

Negative-case analysis was an explicit decision procedure rather than a general statement of openness (Mays and Pope, 2000). When an account complicated a developing interpretation, the full transcript and case synopsis were revisited. Support was not coded as conditional unless an explicit contingency, patterned change in recognition, or clear link between conformity and assistance was present. Material located between open and conditional support remained uncoded as conditional when that threshold was not met. Accounts of parental oversight were re-read alongside cases that framed similar involvement as protection or ambivalent care; mental-health stigma was interpreted alongside emerging openness; and low-pressure relationships were retained as limits on claims about normative enforcement.

These decisions altered the final analysis. P1’s “we are very open” remained an idiographic counter-example within support conditionality; AC3’s “I do not feel very pressured about anything” constrained the adulthood-checklist claim; and negotiated marriage exceptions prevented endogamy from being described as uniformly rigid. Counter-cases were therefore used to narrow definitions, revise theme labels, and specify boundary conditions rather than being mentioned only after the principal interpretation.

### Theories as sensitising lenses, not analytic templates

SIT and FST were used as sensitising lenses: resources for asking interpretative questions rather than categories that participants’ accounts were required to fit. IPA’s double hermeneutic involves the researcher making sense of participants making sense of their experience (Pietkiewicz and Smith, 2014; Smith et al., 2009). Descriptive and linguistic noting therefore preceded higher-order theoretical interpretation. SIT oriented attention to audiences, comparison, belonging, and reputational concern; FST oriented attention to rules, roles, boundaries, alliances, resource distribution, and responses to disagreement. A theoretical interpretation could be retained only when the participant’s language and narrative sequence supplied the mechanism; otherwise it was revised or set aside.

Four safeguards limited theoretical imposition: (1) every interpretative claim required a verbatim anchor and local context; (2) alternative explanations such as care, material necessity, or safety were considered before naming control or homeostasis; (3) counter-cases were preserved in the theme structure, and (4) theoretical claims were separated from descriptive claims about what participants directly reported. This procedure does not eliminate researcher interpretation, but it makes the path from text to concept inspectable.

### Reflexivity and trustworthiness

Reflexive memos documented moments when personal or theoretical assumptions narrowed interpretation (Finlay, 2002). An early tendency to read parental oversight as straightforward control was softened when later cases framed similar involvement as ambivalent care. Earlier transcripts, within-case maps, and cross-case tables were then revisited. Mental-health stigma was similarly interpreted alongside accounts of growing openness so that Mauritian families were not represented as static or uniformly restrictive. An audit trail was maintained through dated memos, analytic notes, case synopses, and versioned theme tables.

Trustworthiness was further supported by returning each transcript to its participant for factual review and minor clarification, extract-led reporting, and independent scrutiny of the interview procedure, thematic coherence, and alignment between quotations and interpretative claims by two qualitative researchers. These practices strengthen credibility and traceability; they do not establish inter-rater reliability or remove the situated role of the researcher (Yardley, 2000).

### Illustrative data use in a theoretical article

The extended excerpts make the analytic difference between single-theory and dual-theory readings visible and show how counter-cases restrict the model. They remain a selected subset of a larger IPA analysis. The article can therefore claim that the framework is capable of producing empirically accountable questions and interpretations in these examples. It cannot claim that the framework has been validated, that the identified processes are prevalent, or that the recursive pathway has been observed longitudinally.

## Part V: scope, current constraints, and future directions

The limitations below constrain the present argument now; they are not merely matters for later research.

*Selected illustrative evidence*. The article develops three theoretically informative sequences from a larger study rather than presenting the full idiographic findings. Selection makes the analytic operation visible but also creates a risk of privileging excerpts that fit the model. The inclusion of counter-cases reduces that risk but does not remove it. Consequently, the present article can demonstrate coherence and empirical accountability, not model validation or thematic prevalence.

*Cross-sectional and retrospective data*. The proposed model is recursive, yet each participant completed one interview. Feedback processes—such as rules loosening after demonstrated responsibility or conflict changing a family’s orientation to a norm—are reconstructed from retrospective narratives rather than observed over time. Causal direction and temporal sequencing therefore remain provisional.

*Small, purposive, English-speaking sample*. The 10 participants provide idiographic depth and variation but cannot support population estimates or systematic subgroup comparisons. English-language interviewing and recruitment through social media and messaging platforms may underrepresent people who are less digitally connected, less comfortable discussing family life in English, or located in more socially isolated circumstances. This narrows the range of Mauritian experiences represented and limits transferability.

*Insufficient basis for intersectional comparison*. Gender, sexuality, class, ethnicity, and religion shape the proposed mechanism, but the design was not powered or structured to compare their independent or intersecting effects. The manuscript can identify father-centred authority, heteronormative scripts, ethnoreligious boundary-making, and material dependence where participants narrated them; it cannot determine which groups face stronger enforcement or how those relations vary across communities.

Incomplete developmental and structural explanation. SIT and FST do not by themselves adequately theorise life-course change, labour and housing markets, migration regimes, or postcolonial class formation. Without these perspectives, the model risks making family dynamics appear static and psychologising constraints that are materially produced. The integrated framework must therefore be used alongside developmental and structural analysis rather than as a complete account of family life (Elder, 1998).

*Context and transferability*. The framework is not claimed to apply universally to contexts described as “collectivist”. Its potential transferability is limited to settings where family decisions are socially visible, intergenerational involvement remains consequential, identity stakes differ across domains, and household members possess unequal capacities to enforce or resist norms. Whether these conditions produce similar processes elsewhere is an empirical question.

Future research should test the proposed links through longitudinal family studies, fuller idiographic reports, and comparative designs across small-island, diasporic, and community-embedded settings. Purposeful recruitment of disconfirming cases—including low-pressure families, non-heteronormative relationships, economically independent adult children, and households with different authority arrangements—would be especially important for refining or rejecting the propositions.

## Conclusion

This article has developed a bounded and power-sensitive integration of Social Identity Theory and Family Systems Theory for analysing how sociocultural norms may enter parent-adult child relationships. SIT identifies how prototypes, comparison, belonging, and anticipated judgement can give an expectation identity and reputational significance. FST identifies the interactional routes through which those stakes may be organised: rules, repeated questioning, alliances, boundaries, emotional tone, and resource allocation. Power analysis specifies who can convert a preference into a consequential household practice, while negative cases identify when the proposed translation does not occur.

The extended illustrations show three distinct operations: support can combine reliable care with approval-contingency but can also remain open; adulthood milestones can become household clocks but can be interrupted by flexible timelines and low-pressure relationships, and marriage can concentrate ethnoreligious and heteronormative stakes while still allowing negotiation, deferral, or exception. These contrasts are central evidence for the framework’s limits, not peripheral qualifications.

The contribution is therefore methodological and analytic rather than confirmatory. The paper specifies a recursive pathway, embeds authority and structural inequality within it, makes the IPA audit trail visible, and states what the selected evidence cannot establish. The framework remains a proposal whose explanatory value depends on fuller empirical testing, continued attention to counter-cases, and use alongside structural and life-course perspectives.

## Data Availability

The original contributions presented in the study are included in the article/supplementary material, further inquiries can be directed to the corresponding author.
